# Draft Genome Sequence of the Ascomycete *Xylaria multiplex* DSM 110363

**DOI:** 10.1128/MRA.00262-20

**Published:** 2020-04-09

**Authors:** Enrico Büttner, Christiane Liers, Anne Richter, Martin Hofrichter, Harald Kellner

**Affiliations:** a Department of Bio- and Environmental Sciences, International Institute Zittau, Technische Universität Dresden, Zittau, Germany; Broad Institute

## Abstract

Xylaria multiplex is an ascomycete fungus that causes soft rot and is often associated with wood. Here, we report a draft genome sequence with an assembly size of 45.6 Mbp, a G+C content of 46.9%, and 10,964 predicted genes, including 617 that encode carbohydrate-active enzymes (CAZymes).

## ANNOUNCEMENT

Xylaria multiplex (Kunze) Fr. 1851 belongs to the ascomycete family Xylariaceae. It is closely related to Xylaria hypoxylon within the so-called clade HY (1) or X2 (2). The species is widespread in various geographic regions and often dwells on decaying hardwood (causing soft rot) but also on bark and leaf litter (1). *Xylaria* spp. are well-known producers of diverse secondary metabolites (3); from *X. multiplex*, multiplolides A and B, two new antifungal 10-membered lactones, were characterized (4). The genome sequence provided in this study may be a resource for biotechnological, pharmaceutical, and ecological studies and will facilitate comparative studies on xylariaceous fungi.

*X. multiplex* DSM 110363 (ribosomal cistron [GenBank accession number MN833802]; proteins RPB2 [KAF2969326], β-tubulin [KAF2963689], and Tef1α [KAF2968635]) was collected on a small piece of wood at the entrance of a humid cave in Bosque Estatal de Río-Abajo in Puerto Rico (18°19′41.5″N, 66°42′57.8″W). It was isolated and cultured on malt-extract agar plates (2%) for biomass production. Genomic DNA was extracted using a standard cetyltrimethylammonium bromide (CTAB)-based protocol and sonographically sheared into 400-bp fragments using an S2 system (Covaris, Woburn, MA, USA). A library was generated using the Ion Plus fragment library kit and sequenced on an IonTorrent S5 system using a 530 chip (Thermo Fisher, Darmstadt, Germany). Altogether, 10.1 million quality-filtered reads (trim function in Geneious Prime 2019.2 [5]; error probability limit, 0.05; trim 3′ end) with an average length of 349 bp were used for *de novo* assembly using MIRA 4.0 (minimum reads per contig, 100) (6). A second assembly step using the assembler of Geneious Prime 2019.2 joined overlapping and filtered duplicate contigs and resulted in 389 contigs (maximum read length of 1,020,413 bp). The final assembly had a G+C content of 46.9% and a total size of 45.6 Mbp. Assembly quality was verified using QUAST v5.0.2 (7) and resulted in an *N*_50_ value of 241,994 bp and *L*_50_ of 57. Genome single-copy ortholog analysis performed with BUSCO v3 (predictor, Aspergillus oryzae; fungal data set, Ascomycota_*odb9*) (8) reported a genome completeness of 96.4%. Gene prediction was performed using the AUGUSTUS v3.2.2 Web server (predictor, Aspergillus oryzae) (9) and resulted in 10,964 protein-coding genes, which were compared and annotated using the nonredunant (nr) database (NCBI). A carbohydrate-active enzyme (CAZyme) search was performed using dbCAN (HMMdb v7; E value, <1e^−15^; coverage, >0.35) (10) and identified 617 enzymes, which are categorized into 271 glycoside hydrolases, 87 carbohydrate esterases, 15 polysaccharide lyases, 80 glycosyltransferases, and 138 enzymes with auxiliary (oxidative) activities. Furthermore, 26 carbohydrate-binding modules (CBMs) were found, including 10 CBM1 modules associated with cellulose binding. Some enzymes putatively related to the modified lignocellulose and aromatics, such as 2 cellobiose dehydrogenases, 11 multicopper oxidases (laccases), 5 unspecific peroxygenases, 3 dye-decolorizing peroxidases, and 2 generic peroxidases, were manually annotated and are available under the GenBank accession numbers shown in Table 1. Secondary metabolite biosynthetic gene clusters (BGCs) were predicted using antiSMASH v5.0.0 (11). A total of 53 BGCs were identified, including BGCs for the production of 29 polyketides, 7 nonribosomal peptides, 10 terpenes, 1 indole cluster, and 1 posttranslational modified peptide.

**TABLE 1:** CAZyme classes and enzymes of interest detected in the genome of DSM 110363

Enzyme or domain group	No. of proteins	GenBank accession no.
Glycoside hydrolases	271	
Glycosyltransferases	80	
Polysaccharide lyases	15	
Carbohydrate esterases	87	
Auxiliary activities	138	
Associated modules		
Carbohydrate-binding modules	26	
Cellulose-binding domain CBM1	10	
Enzymes of interest		
Unspecific peroxygenases	5	KAF2962640, KAF2971576, KAF2970693, KAF2967605, KAF2966492
Multicopper oxidases (laccases)	11	KAF2963317, KAF2963168, KAF2972509, KAF2971891, KAF2971316, KAF2973423, KAF2966961, KAF2965160, KAF2963826, KAF2963738, KAF2968172
Generic peroxidases (class II-related)	2	KAF2971615, KAF2969393
Dye-decolorizing peroxidases	3	KAF2971550, KAF2971125, KAF2964055
Cellobiose dehydrogenases	2	KAF2971080, KAF2966942

### Data availability.

This whole-genome shotgun project has been deposited at DDBJ/ENA/GenBank under the accession number WUBL00000000. The version described in this paper is the first version, WUBL01000000. The Sequence Read Archive (SRA) number is SRR10723780. All referenced genes are cited within BioProject number PRJNA596119.
